# Urban malaria transmission in a non-endemic area in the Andean region of Colombia

**DOI:** 10.1590/0074-02760170113

**Published:** 2017-12

**Authors:** Pablo E Chaparro, Karen Molina, Alberto Alzate, Julio Padilla, Myriam Arévalo-Herrera, Sócrates Herrera

**Affiliations:** 1 National Institute of Health of Colombia, Bogotá, Colombia; 2 Malaria Vaccine and Drug Development Center, Cali, Colombia; 3 Caucaseco Scientific Research Center, Cali, Colombia; 4 Ministry of Health and Social Protection of Colombia, Bogotá, Colombia; 5 Universidad del Valle, School of Health, Cali, Colombia

**Keywords:** malaria, urban population, migration, Plasmodium, Colombia

## Abstract

**BACKGROUND:**

Rapid urbanisation in difficult socio-economic conditions such as inadequate housing infrastructure, lack of public services, improper sanitation, and poor water drainage systems in vegetation-rich areas lead to ecological conditions that are conducive to the breeding of mosquitoes and transmission of malaria, in semi-urban and urban settings.

**OBJECTIVES:**

This study aimed to describe the cases of malaria that were reported in the peri-urban areas of Pereira (Colombia), between 2008 and 2015.

**METHODS:**

A retrospective study was conducted using data from the Malaria Surveillance System 2009-2015 and an outbreak study (between December 2008 and March 2009). Frequency distributions and summary measures, as well as univariate analysis were performed for all the variables in consideration. The annual parasite index (API) was calculated.

**FINDINGS:**

Data on 214 cases were obtained from the surveillance system. A majority of the cases were reported in men (63.1%), followed by in children < 15 years (23.8%), and were caused predominantly by *Plasmodium vivax* (86.0%), with most of the infection occurring in the urban areas (52.8%) of Pereira. The API, by sex and age group, was higher among men ≥ 80 years. The outbreak study reported 14 cases of malaria in rural/peri-urban neighborhoods, and it was observed that the anopheline breeding sites were in close proximity to the houses in these areas. This population did not use protective measures against mosquitoes and chemical control was conducted through residual and spatial insecticide spraying.

**MAIN CONCLUSIONS:**

This study suggested the presence of autochthonous malaria transmission, in Pereira, between 2008 and 2015, most of which were cases of *P. vivax.* A greater intensity was observed between 2008 and 2009 when malaria was possibly reintroduced to the region. During the years of the study, a gradual decrease in the number of reported cases of malaria was observed in Pereira, except for the time period between 2008 and 2009 when a spike was noted (estimated using the API); this was most likely caused by an outbreak. Interventions that are more aggressive in nature are required to prevent further malarial transmission and dissemination.

The global malaria incidence decreased from 271 million cases in 2000 to 212 million in 2015, corresponding to a decline of 21.8%. In the American continent, the decrease was much more significant, going from 1.2 million cases in 2000 to 8,000,000 in 2015 (33.3%) (WHO 2016). Despite this significant decrease, malaria remains a threat to communities in the tropical and subtropical areas of the world, where it imposes a significant burden and has an associated economic impact.

While there has been significant progress in the field of malaria elimination in some endemic countries, it appears that in many others, a variety of factors are impeding the expected decrease in the incidences of malaria (Méndez & Carrasquilla 1995, Ochoa & Osorio 2006, Rodríguez et al. 2011). Rapid urbanisation combined with difficult socio-economic conditions such as inadequate housing infrastructure, lack of public services, improper sanitation, and poor water drainage systems in vegetation-rich areas create ecological conditions that are conducive to mosquito breeding and malaria transmission. In addition to the immigration of infected individuals to urban and peri-urban areas, it is to be noted that mosquito reservoirs already exist even in the more developed regions, in which the residing populations have no immunity to malaria (de Castro et al. 2004). Climate change seems to influence malaria transmission too, and it appears that malaria vectors are adapting to higher altitudes (Siraj et al. 2014).

During the last 10 years, a significant decrease in the malaria transmission rates has been observed in Colombia (Buitrago et al. 2013, Chaparro et al. 2013, Forero et al. 2014); simultaneously, however, reports on the presence of urban malaria transmission have surfaced. A total of 17 locations were recently reported as probable urban and peri-urban malaria transmission sites (Padilla et al. 2015). Although most of these sites correspond to urban settings within endemic regions, with poor socio economic development, some of them are present in the more developed areas of the country, with overall better sanitation, economic conditions and a higher population density. Risaralda is in the Andean region of Colombia, and has a significantly higher socio-economic standard than that of the whole of the Pacific coast region, which is the most endemic region (in terms of malaria) in the country. However, Risaralda - in particular, its capital city Pereira - experiences a high migration rate, with individuals from malaria-endemic regions, especially Chocó (one of its neighbouring departments), migrating to this coffee-growing region. In the past several years, most of the cases of malaria notified in Risaralda have been recorded in Pueblo Rico and Mistrató, which are towns located between Chocó and Pereira (Rodríguez et al. 2012). In 2013, ~49% of the malaria cases in Pereira were recorded as having originated in Chocó, and the remaining cases, originated in the municipalities of Antioquia and Risaralda. The eco-epidemiological conditions of Pereira such as its altitude (< 1,500 m), temperature (18-28°C) (Paaijmans et al. 2010, Mordecai et al. 2013, Croft 2014), rainfall and the different uses of the land, which lead to the creation of water collection sites, support the establishment of permanent breeding sites for different *Anopheles* species, which altogether favour malaria transmission. At least six mosquito species with malaria transmission potential: *Anopheles pseudopunctipennis, An. darlingi, An. lepidotus, An. punctimacula, An. argyritarsis, An. apimacula* and *An. eiseni* are present in this region (Olano et al. 2001). The latter five species *(An. lepidotus, An. punctimacula, An. argyritarsis, An. apimacula and An. Eiseni)* has been found at altitudes > 1,200 m (from Cerritos to Rocio Bajo, in El Poblado) as well as in more low-lying areas (900 m above sea level, in the districts of Caimalito and Puerto Caldas). In addition, Pereira's proximity to La Virginia, one of its neighbouring municipalities, where there is a continuous movement of people to and from Chocó, could also contribute to malaria transmission, establishment and maintenance. The aim of this study was to describe the characteristics of rural and urban malaria cases reported in Pereira, between 2008 and 2015, and to obtain evidence to support malaria surveillance policies for regions which have sub-optimal conditions for malaria transmission.

## MATERIALS AND METHODS

*Type of study* - This was a descriptive, observational study of malaria cases between 2008 and 2015, and a field research on an outbreak of malaria that occurred between December 2008 and March 2009, in Pereira. This study used information from the Colombian National Surveillance-SIVIGILA.

In Colombia, after a malaria case is diagnosed, it must be reported to the SIVIGILA either through reporting units (RU) or primary data generating units (PDGU). RUs correspond to “primary agents” (microscopists), who are responsible for the diagnosis, treatment and notification, while PDGUs correspond to public or private health services, in which doctors are responsible for these tasks. The diagnosis of infection is then confirmed by the thick blood smear (TBS) or rapid diagnostic test (RDT). The information provided by the RUs and the PDGUs is sent to the municipal health secretary, and thereafter to the departmental health secretary and finally to the National Health Institute. At each of the levels in which the surveillance information is received, it is submitted to processes that ensure its quality, so that it is immediately analysed and serves as a basis for decision-making.

**Fig. 1:**
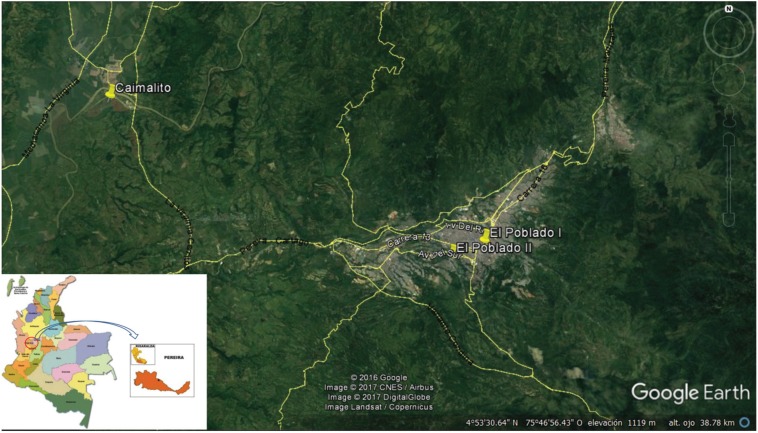
study area in Pereira, in the Risaralda department, in Colombia. Source: Google Earth (accessed April 20th 2017).

*Study area* - The study sites of the outbreak that occurred between December and March (2008-2009) were the Carbonera village, located in Caimalito, in rural Pereira, as well as the Poblado I and Poblado II neighbourhoods, that correspond to the urban area of Pereira. The sites of the cases reported between 2008 and 2015 were in the municipality of Pereira, which is the capital of Risaralda - one of the 32 administrative departments in the country (Fig. 1). Pereira is located in the central Andean mountain range, with an altitude of 1,411 m, occupying an area of ~ 609 km^2^ (CARDER 2013), an average annual temperature of 22°C and an average annual rainfall of 2,750 mm^3^. In 2015, the total number of residents of the city was estimated to be 4,69,644. The region's river system is composed of the Cauca, Barbas, La Vieja, Otún and Consota rivers, with numerous tributaries. The Poblado-I and Poblado II neighbourhoods are located in the Cafetal area, which is a flood zone of the Consota river. The vegetation here predominantly comprises Musacea and Mimosacea species, which makes it particularly prone to the presence of malaria mosquito vectors. In 2008, this region was found to have a population of 2,669 people, and was characterised by poor socio-economic conditions. Poblado-II is adjacent to Poblado-I, and has a population of 4,283 inhabitants. This neighbourhood has a better quality of life and is located approximately 2 km from the central part of Pereira's urban area. Caimalito is located in the Carbonera village, in the Northwestern rural area of Pereira, with a population of 8,007 inhabitants (as of 2008) who live under unfavourable socioeconomic conditions. It is located approximately 25 km from the central part of Pereira's urban area, and provides an environment that it ideal for malaria transmission, as it is a transit zone for people from endemic areas.

*Case definition* - A confirmed case of malaria confirmed was defined as a patient with a current or recent (up to two weeks prior) febrile episode (> 37.5°C), in endemic areas in the last 15 days, with a confirmed diagnosis, based on the identification of a *Plasmodium* species in a parasitological test (Giemsa stained TBS or RDTs), according to the malaria public health surveillance.

An autochthonous case was one in which malaria was locally contracted, and an imported case was defined as that in which the disease was acquired outside the specific area in which it was found (Snow & Gilles 2002).

*Descriptive variables* - Data from the surveillance system were used, and the variables considered were: number of malaria cases per year, parasitic species, area of origin/occurrence of the case (urban, rural), age, sex, type of social security affiliation, ethnicity, date of the onset of symptoms, date of consultation, precipitation, humidity and temperature. For the outbreak analysis, the aforementioned variables, as well as the occupation and case classification (autochthonous or imported) were included.

A typical urban area in Colombia consists of groups of buildings and contiguous structures grouped into blocks, which are delimited by streets or avenues, with a number of essential services such as aqueducts, sewage systems, and electrical energy, as well as hospitals and schools. The capital cities and the remaining municipal administrative cities are considered urban areas. In contrast, a rural area is characterised by the dispersed disposition of houses and agricultural holdings, and a lack of road structure and public services. A peri-urban area is one that combines the characteristics of both urban and rural areas, and is usually located outside the city.

*Field research and origin of the cases in the 2008-2009 outbreak* - The first case of a patient with symptoms in Poblado-I was reported to have undergone a consultation in December 2008; two other cases appeared - one in December and the other in January 2009, and one of these cases occurred in the house of the first patient. These three cases corresponded to *Plasmodium falciparum* infection. Simultaneously, at the end of December 2008, in Caimalito, which is approximately 22 km away from Poblado I, a case of *P. vivax* infection was reported. Thereafter, 10 more cases of *P. vivax* infection were reported in the same area. All the cases were tested by microscopy using TBS, at the time of the consultation.

The survey included an epidemiological study to confirm the primary source of infection, using active case detection (ACD); the geographical limitation of the transmission foci and entomological characterisation; and activities pertaining to the diagnosis, treatment, promotion, prevention and control (epidemiological alert). The entomological research was conducted in March 2009, and consisted of an indoor and outdoor search for adult mosquitoes, in the houses of the individuals who were reported as infected, and larvae collection from the breeding sites located adjacent to these houses. The taxonomic identification was performed using morphological keys (Tinker 1982, González & Carrejo 2009).

*Statistical analysis* - Frequency distributions and summary measures were performed using the Tableau software version 6.0. Univariate analysis was performed for all the considered variables. The API was then calculated.

*Ethics* - This study was carried out in accordance with the Helsinki Declaration of 1983.

The study protocol was evaluated and approved by the Ethical Committee of Centro Internacional de Vacunas (CECIV: DMID Protocol Number: 14-0097).

## RESULTS

**Fig. 2:**
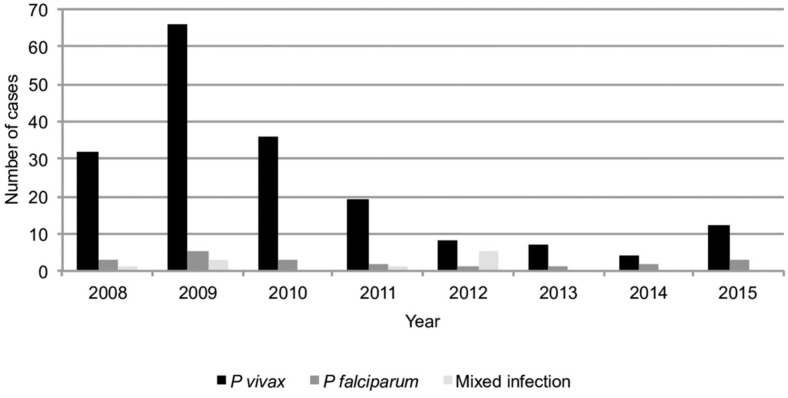
malaria cases by parasitic species. Pereira, 2008-2015.

**Fig. 3:**
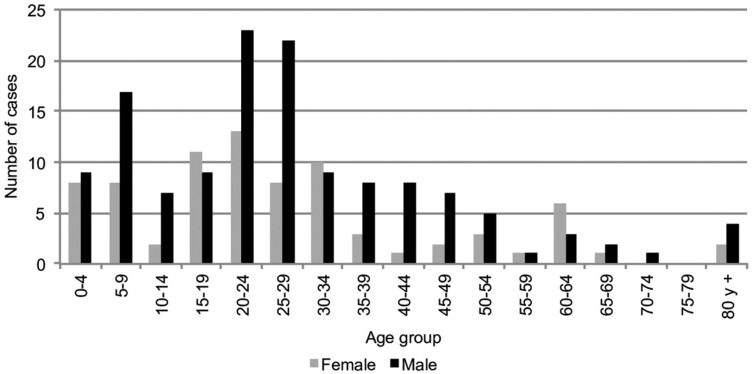
malaria cases by gender and age group. Pereira, 2008-2015.

A total of 214 cases of malaria were reported in Pereira, between 2008 and 2015, and a peak (74 cases) was observed in 2009 (Fig. 2). Of these cases, 135 (63.1%) of those infected were men. *P. vivax* infection was the most prevalent [184 (86.0%)], followed by *P. falciparum* infection [20 (9.3%)]. Ten of these cases (4.7%) corresponded to mixed infections. The average age of the patients was 27.8 years. Importantly, 51 (23.8%) of these cases were reported in children < 15 years, and 17 (33.3%) of these were in the 0-4 years age group (Fig. 3).

By sex and age group, the incidence of malaria was highest among men aged 20 to 24 years, and 25 to 29 years (Fig. 3), and the ratio of the incidences of *vivax: falciparum* was 9:1. The highest API was observed in men aged > 80 years (0,18/1000 men), followed by in men aged 25 to 29 years (0,15/1000 men) and then those aged 20 to 24 years (0,15/1000 men). Among females, the highest API was observed in the 20-24 years age group. Only 51 of these cases among men (37.8%) were consulted through a microscopists within the first 48 h after the onset of symptoms.

**Fig. 4:**
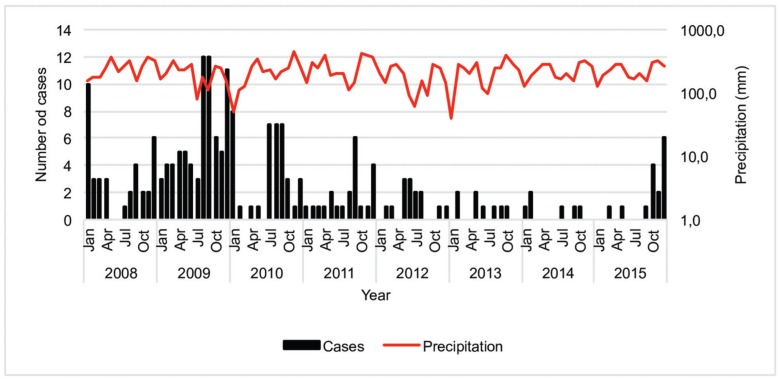
epidemiological curve of malaria cases. Pereira, 2008-2015.

In the period between 2008 and 2015, the relative humidity was between 53.3 and 85.7%, temperature was between 19.5 and 23.5°C and precipitation was 39.8 to 453.6 mm. The increase in the incidences of malaria, in August and September 2009, occurred after a rainy period (Fig. 4). A total of 113 cases (52.8%) were notified in the urban area and 101 cases (47,2 %) in the rural areas; 75.7% of the patients were registered in the social security system. Most such cases (184; 86.0%) occurred in the white/mestizo population, 18 (8.4%) of these cases involved Afro-Colombians and 12 (5.6%) cases involved individuals of indigenous origin. The median time between the onset of symptoms and diagnosis was four days (range 0 days − 65 days); 20.1% of these individual cases were diagnosed in the first 48 h and 57.9% after 96 h after the onset of malaria symptoms (Table I), despite 18 cases (half of which cases of *P. vivax* infection) displayed severe malaria characterised by pulmonary dysfunction (seven cases), as well as hematological (five cases) and liver (four cases) abnormalities. Of these cases, 8 (44.4%) involved individuals aged 20 to 29 years old, three (16.7%) involved those aged 0 to nine years old, two involved those aged 10 to 19 years old, and nine involved those who were older than 29 years.

**TABLE I:** Time between onset of symptoms and date of consultation of malaria cases by *Plasmodium* species in Pereira 2008-2015

Time between onset of symptoms and consultation	*P. vivax*		*P. falciparum*		Mixed infection		Total	
	(n)	(%)	(n)	(%)	(n)	(%)	(N)	(%)
24 h	38	20.7	3	15.0	2	20.0	43	20.1
48 h	21	11.4	4	20.0	3	30.0	28	13.1
72 h	18	9.8	1	5.0		0.0	19	8.9
≥ 96 h	107	58.2	12	60.0	5	50.0	124	57.9
	184	100	20	100	10	100.0	214	100

**Fig. 5:**
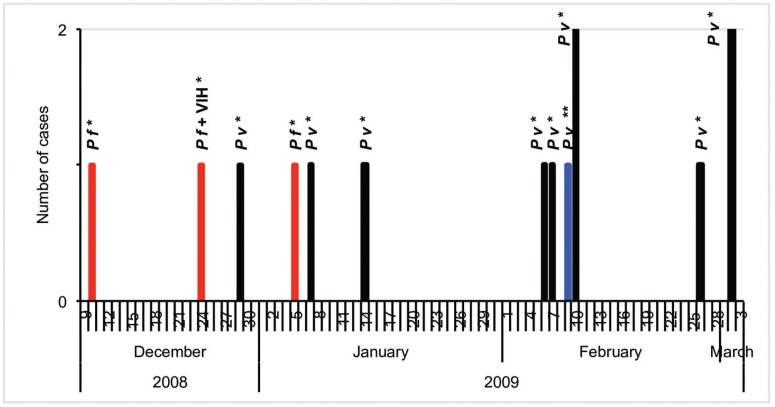
monthly distribution of malaria cases in Pereira, between epidemiological weeks 49 of 2008 (9th December) and 12 of 2009 (3rd March).

Between December 2008 and March 2009, 14 cases of malaria were reported in the study area; this was considered a malaria outbreak. Eight of these cases occurred in women with an average age of 22.2 years. Eleven of these cases corresponded to *P. vivax* infection and the remaining to *P. falciparum* infection (Fig. 5); none of the infected individuals had a history of travel to malaria-endemic areas. A 45-year-old patient was diagnosed with HIV (Table II). Three cases were caused by *P. falciparum*, all of which qualified as autochthonous cases - two of these cases occurred in Poblado I, with a time gap of 25 days; one case occurred in Poblado II, 14 days after the first case presented in Poblado I. Eleven cases were caused by *P. vivax*, of which one was cataloged as an imported case. A 9-day gap was observed between the two autochthonous cases, and in relation to the case imported 39 days elapsed. In the case of *P. vivax* infection, an average of three days was observed between the onset of symptoms and the consultation of a doctor. In the case of *P. falciparum,* the infection presented an average of 7.7 days prior to the consultation. Antimalarial treatment was provided immediately after the malaria diagnosis was confirmed and all the infected individuals recovered.

**TABLE II:** Demographic and epidemiological characteristics of malaria cases in Pereira, Colombia, between epidemiological weeks 49 of 2008 (9th December) and 12 of 2009 (3rd March)

Characteristics		(n)	(%)
Sex	Male	6	42.9
	Female	8	57.1
Age	Mean age	22.2 (SD: 19,9)	
Occupation	Smith	1	7.1
	Sewing machine operator	1	7.1
	Mechanical, motor vehicles and related	1	7.1
	Pensioner	1	7.1
	Home (housewife)	2	14.3
	Student	3	21.4
	Minor	5	35.7
Origin of cases	Caimalito	10	71.4
	Poblado-I	2	14.3
	Poblado-II	1	7.1
	Segovia (Antioquia)	1	7.1
*Plasmodium* Species	*Plasmodium vivax*	11	78.6
	*P. falciparum*	3	21.4
Ethnicity	African American	1	7.1
	White/Mestizo	13	92.9

SD: standard deviation.

Entomological surveys conducted in the different villages indicated that these populations did not use basic mosquito protection measures such as doors, windows or bed nets. No adult mosquitoes were captured, as the sanitary authorities identified the breeding sites in January 2009, in Caimalito, and chemical intervention was carried out, with the use of residual and spatial insecticide spraying. In addition, the rainfall conditions in March 2009 did not allow to identify active anopheles breeding grounds. However, there were four mosquito breeding sites adjacent to the residences of the infected patients, in Caimalito - in only one of these, six anopheline larvae were captured, but they were found to be *Anopheles (Nyssorhynchus) argyritarsis* in the immature stages. No larvae were found in the other productive ponds, due to the intense rains during the field research. In the Poblado sector, seven breeding sites were inspected, but none were of an anopheline nature. Indoor and outdoor insecticide spraying was performed in all the affected houses.

## DISCUSSION

This study suggested the presence of autochthonous malaria transmission in Pereira, between 2008 and 2015; most of these cases were observed to be caused by *P. vivax,* and a greater intensity was recorded between 2008 and 2009. During the years of the study, a gradual decrease in the number of cases of reported malaria was observed; however, between 2008 and 2009, the API was found to be high, most likely due to an outbreak. Pereira is one of the municipalities in the “Colombian coffee culture region” that has historically reported sporadic incidences of malaria, as the ideal conditions for coffee growth correspond to an altitude of 1,411 m and a temperature of ~ 22°C. The five departments in this region did not seem to be receptive to malaria although their ecological conditions are considered borderline for transmission. Due to the higher standard of living and better health services in the urban areas of Pereira, many patients are referred to this city for medical attention; therefore, even if they were reported in Pereira, malaria cases most likely originated in the rural areas of the region.

According to the results presented, 75.7% of the patients were in the social security system. Not all the patients were diagnosed at health service centres, and a high percentage was diagnosed by malaria microscopists but had no social security. The Colombian social security system does not show universal coverage (92.8% coverage in 2015) (DANE 2016).

Urban and peri-urban malaria have been reported before in Colombia. A recent study showed that 17 malaria-endemic municipalities reported 18,113 urban and peri-urban cases that occurred in 2008 and 2012 (Padilla et al. 2015). However, it is likely that at least some of the cases did not originate in urban settings but at the temporary housing of patients seeking diagnosis in urban health service centres. One of the main confounders of malaria notification is the confusion between “site of origin”, “place of residence” and “case report site”, possibly leading to a wrong classification, with regards to the site of infection. Urban malaria has also been reported based on the results of epidemiological and entomological analyses of local autochthonous outbreaks (Buitrago et al. 2013, Quintero et al. 2015), associated specially with indigenous people who lived in clusters or people who had a history of displacement due to armed conflict.

The analysis of malaria cases in the December 2008 to March 2009 outbreak in Poblado and Caimalito suggests autochthonous transmission in those areas. These cases shared common transmission characteristics: susceptible individuals, confined to one place, a history of lack of travel to endemic areas, no history of previous exposure, and the presence of favourable environmental conditions for parasite development and positive mosquito breeding sites. Although it was not possible to confirm the presence of infected female anophelines, to establish a real causal relationship with malaria cases, other positive factors for malaria transmission, including the confirmed diagnosis of malaria in urban health service centres, confirmed infection in urban residents, competent vectors and climate change, which appears to influence malaria transmission in higher altitudes (Siraj et al. 2014), suggest autochthonous transmission. Although we cannot conclusively define the origin of the malaria cases reported from 2008 to 2015, it appears that most of these cases may be autochthonous in nature. The cases of *P. falciparum* infection in Poblado I and Poblado II were considered autochthonous, in the absence of antecedents that indicated an epidemiological connection. Likewise, all the cases of *P. vivax* infection in Caimalito were considered autochthonous, except for one which was classified as imported and was preceded by five cases.

In 2013, the Secretary of Health of Pereira reported sporadic and isolated cases from Poblado I, Poblado II and Caimalito, which were classified as autochthonous. However, as Pereira has a constant transit of people from endemic areas (Martens & Hall 2000, Jitthai 2013) including Chocó, which is among the most malaria-endemic regions of Colombia. It is highly likely that individuals with *Plasmodium* spp asymptomatic infections had visited the area which due to the presence of malaria vectors became foci of infection; in this study, we did not detect asymptomatic cases using TBS or RDT. Symptomatic cases could maintain low parasitaemia (submicroscopic) for long periods without treatment, therefore becoming the origin of new clinical cases that would represent malaria outbreak (Mabuza et al. 2001, Govere et al. 2002).

The presence of *Anopheles argyritarsis* has been reported in La Virginia (in Risaralda), which is one of Pereira's neighbouring municipalities (Quintero et al. 2015). This mosquito species grows in pools of rain water that accumulate in the soil and sometimes in artificial containers such as cans and water troughs for animals (Galante et al. 2014). The habitats of the immature forms are characterised by little herbaceous vegetation and are generally located in areas of secondary growth (Linthicum 1988, Montoya-Lerma et al. 2011). Although this species is not considered a primary vector for malaria transmission, it may be important when present at high densities. Even though it is rarely found inside houses, and only feeds sporadically on humans, malaria parasites have been identified in *An. Argyritarsis,* in its habitat (Faran 1980).

This study had several limitations. Firstly, there was no clear evidence on the presence of infected anopheline vectors in the areas studied, for the establishment of a causal relationship with malaria cases. Secondly, there were mistakes in the processing of mandatory malaria reporting forms, probably due to the lack of training or proper instructions. In addition, it is to be taken it consideration that the residence or notification site of a malaria case was not necessarily the infection site. Finally, there was a lack of epidemiological studies on autochthonous cases reported sporadically by the Secretary of Pereira, since 2008.

Despite these limitations, the results presented herein support previous preliminary reports of malaria in urban and peri-urban settings (Wilson et al. 2015). There is currently a global effort to clarify the definition of urban malaria and to understand the influence of imported malaria on the reporting of cases in urban areas. Additionally, this study questions the reliability of the origin of cases in official report forms and points to a need for improving the classification with an exact site of infection and differentiation in terms of whether a case is urban or peri-urban in occurrence. The present study also points to the need for epidemiological studies of cases that are classified as autochthonous, and their entomological correlations, especially in areas such as Caimalito which have mixed urban and rural characteristics.

The climate, presence of vectors, and the influx of people from endemic areas such as Chocó, make Pereira and its surroundings vulnerable to malaria. The development of a plan that includes the specific recognition of the site of infection through an appropriately used mandatory report form is required. Future epidemiological studies are also required to understand cases of urban malaria, outside of the context of an urban outbreak.

In response to malaria outbreaks in very low transmission areas, it is recommended that prevention and control measures be maintained by the health authorities; these include active case detection for early diagnosis and timely treatment, vector control measures and community education activities.
